# Prevalence of Homologous Recombination Deficiency in First-Line PARP Inhibitor Maintenance Clinical Trials and Further Implication of Personalized Treatment in Ovarian Cancer

**DOI:** 10.3390/cancers15123095

**Published:** 2023-06-07

**Authors:** E Sun Paik, Ha Kyun Chang, Sanghoon Lee

**Affiliations:** 1 Department of Obstetrics and Gynecology, Kangbuk Samsung Hospital, Sungkyunkwan University School of Medicine, Seoul 03181, Republic of Korea; espaik@naver.com; 2 Department of Obstetrics and Gynecology, Korea University Ansan Hospital, 123, Jeokgeum-ro, Danwon-gu, Ansan-si 15355, Republic of Korea; 3 Department of Obstetrics and Gynecology, Korea University Anam Hospital, 73, Goryeodae-ro, Seongbuk-gu, Seoul 02841, Republic of Korea

**Keywords:** PARP inhibitors, ovarian cancer, *BRCA*, HRD, prevalence

## Abstract

**Simple Summary:**

The therapeutic effect of Poly-ADP-ribose polymerase (PARP) inhibitor has been demonstrated in ovarian cancer patients with *BRCA* mutation or homologous recombination deficiency (HRD). HRD analysis at diagnosis determines treatment eligibility in ovarian cancer. In classifying the HRD patient group, different results may be observed depending on the test methods, and evidence of the possibility of differences in HRD prevalence between races was shown through representative clinical trial results. Accordingly, we are going to suggest the influence of individual treatment for ovarian cancer.

**Abstract:**

Among ovarian cancer patients with *BRCA* mutation or homologous recombination deficiency (HRD), the efficacy of Poly-ADP-ribose polymerase (PARP) inhibitors such as olaparib, niraparib, veliparib, and rucaparib has been proven in a number of clinical trials. *BRCA* mutation and HRD are currently indicated for PARP inhibitor maintenance treatment in ovarian cancer. HRD diagnostic tests examine various components, resulting in different HRD status definitions and, as a result, different treatment decisions. A number of HRD diagnostic tests exist, but test results provided by different companies may differ as they use different methods and different cutoffs. HRD prevalence difference was shown between PARP inhibitor maintenance trials. It is important to select an appropriate method that can present accurate HRD phenotypes to predict sensitivity to PARP inhibitors so that patients who are most likely to benefit from treatment are selected. Additionally, in the subset data of the PARP inhibitor maintenance trials, there was a difference in HRD prevalence by race as higher HRD prevalence in Japanese and Chinese ovarian cancer patients was shown. Further large-scale investigations on racial differences in HRD prevalence are needed and this may contribute to changes in determining the treatment plan and personalized treatment in ovarian cancer patients.

## 1. Introduction

Ovarian cancer has the poorest prognosis and the highest mortality among gynecological cancers [1]. Cytoreductive surgery and platinum-based chemotherapy are traditional standard treatment methods [2], and additional studies have reported that the use of the angiogenesis inhibitor bevacizumab increases progression-free survival (PFS) in high-risk groups [3]. Aggressive surgery, chemotherapy, and targeted therapy are applied to ovarian cancer treatment, but 70–75% of ovarian cancer patients still experience recurrence, and the 5-year survival rate is only 23%. There are still needs for additional treatment for ovarian cancer patients.

Poly-ADP-ribose polymerase (PARP) inhibitors are anti-cancer drugs, and the effectiveness of first-line maintenance therapy in the treatment of ovarian cancer patients has recently been proven through several studies [4,5,6,7]. Among ovarian cancer patients with *BRCA* mutation or homologous recombination deficiency (HRD), the efficacy of PARP inhibitors has been proven, and *BRCA* mutation and HRD are currently indicated for PARP inhibitor maintenance treatment. Approximately 11–15% of ovarian cancer patients have *BRCA1/2* germline mutations, 7% have *BRCA1/2* somatic mutations, and it has been reported that HRD is found in about 50% of patients with epithelial ovarian cancer [8,9]. Through the HRD prevalence shown in various randomized clinical trials (RCTs) and other research results, we will investigate a difference in prevalence between specific patient groups and discuss further the direction in which this part will affect personalized treatment in ovarian cancer patients.

## 2. HRD in Ovarian Cancer and PARP Inhibitor

Recently, HRD status has been proven as an important biomarker in ovarian cancer treatment with predictive and prognostic value. Approximately 50% of high-grade serous ovarian cancer is HRD according to the Cancer Genome Atlas (TCGA) project [10]. HRD is a complicated genomic signature that appears when cells cannot repair damaged double-stranded DNA via homologous recombination repair (HRR) pathway [11]. For maintaining genomic stability and cell function, cells must repair DNA damages. The compromised HRR pathway may lead to genomic instability in the form of genomic scarring, resulting in malignant transformation [12]. *BRCA1* and *BRCA2* as well as *ATM*, *BARD1*, *BRIP1*, *H2AX*, *MRE11*, *PALB2*, *RAD51*, *RAD51C/D*, *RPA* and Fanconi Anemia Complementation Group genes are representative genes that have important roles in the HRR pathway as important causative factors of HRD [13]. HRD-related genomic markers, known as “scars”, can be explained as abnormalities that cause structural modifications in chromosomes. The most substantial genomic scars are loss of heterozygosity (LOH) [14], telomeric–allelic imbalance (TAI) [15], and large-scale state transitions (LSTs) [16], which are components for assessment of the genomic instability score (GIS) to inform the status of HRD.

Actions of PARP inhibitors are based on synthetic lethality in HRD-positive tumor cells. PARP1 is an enzyme associated with the recovery of single-strand DNA breaks via the base excision pathway [17]. PARP inhibitor binds to PARP1 at single-strand DNA breaks to avoid effective repair and to cause DNA-protein crosslinks processing into double-strand breaks (DSB), which leads to increased genomic instability and cell death in *BRCA1/2*-mutated or other HRD-related cells that are defective in their DSB repair functions. Based on these findings, HRD has been identified as a prognostic biomarker for PARP inhibitor therapy in ovarian cancer, and other malignancies such as breast, pancreatic, and prostate cancer [18,19,20].

## 3. First-Line Maintenance PARP Inhibitor Treatment RCTs in Ovarian Cancer

The efficacy of PARP inhibitor as first-line maintenance therapy was shown in a number of clinical trials. In a phase 3 trial SOLO-1/GOG-3004/ENGOT [4], a total of 391 ovarian cancer patients with *BRCA* mutation (germline or somatic) were enrolled for efficacy of olaparib maintenance therapy. The median PFS for the olaparib group was 49.9 months, compared to 13.8 months in the placebo group with 70% reduced risk (hazard ratio [HR] 0.3; 95% confidence interval [CI] 0.23–0.41; *p* < 0.001). The SOLO-1 [4] was a representative study that marked the beginning of PARP inhibitor first-line maintenance in ovarian cancer patients, but it was conducted only for ovarian cancer patients with *BRCA* mutation. Information for HRD and *BRCA* wildtype ovarian cancer patients could not be provided. In the other trials using PARP inhibitor maintenance in ovarian cancer patients, the analysis was conducted for all patients and HRD was used as a criterion for stratification. Table 1 summarizes the results of trials with HRD prevalence data using PARP inhibitors as first line maintenance in ovarian cancer patients. These studies showed the efficacy of PARP inhibitors in the HRD population as well as the overall population.

In a phase 3 trial of PRIMA/ENGOT-OV26/GOG-3012 [5], niraparib was investigated as a first-line maintenance therapy in patients with advanced-stage ovarian cancer patients regardless of *BRCA* status. A total of 733 ovarian cancer patients underwent randomization to receive niraparib or a placebo up to a 36-month period. In 373 patients with HRD, the median PFS was 21.9 months in the niraparib maintenance group compared to 10.4 months in the placebo group (HR 0.43; 95% CI 0.31–0.59; *p* < 0.001). In the overall population, the niraparib maintenance group had a median PFS of 13.8 months, compared to 8.2 months for the placebo group (HR 0.62; 95% CI 0.50–0.76; *p* < 0.001).

In the phase 3 VELIA/GOG-3005 [6] trial, veliparib as a maintenance treatment and alongside chemotherapy was evaluated. In this study, 1140 patients who had newly diagnosed advanced ovarian cancer were randomized in a 1:1:1 ratio. They were assigned to one of three treatment groups: chemotherapy plus placebo followed by placebo maintenance (control), chemotherapy plus veliparib followed by placebo maintenance (veliparib combination only), or chemotherapy plus veliparib followed by veliparib maintenance (veliparib throughout). The results showed that the median PFS in the veliparib throughout group was 23.5 months, which was significantly longer than the median PFS of 17.3 months in the placebo throughout group (HR 0.68; 95% CI 0.56–0.83; *p* < 0.001). In the *BRCA*-mutation cohort and HRD cohort, the veliparib throughout group showed a prominent reduced risk (HR 0.44; 95% CI 0.28–0.68; *p* < 0.001, and HR 0.57; 95% CI 0.43–0.76; *p* < 0.001, respectively).

In the PAOLA-1/ENGOT-OV25 trial [21], olaparib combined with bevacizumab as first-line maintenance in ovarian cancer patients was investigated. A study investigated 806 patients with newly diagnosed, advanced, high-grade ovarian cancer who had responded to platinum-taxane and bevacizumab. All participants received maintenance bevacizumab every 3 weeks for 15 months, and were randomized in a 2:1 ratio to receive either olaparib or placebo. The results showed that the median PFS for the olaparib/bevacizumab group was 22.1 months, which was significantly longer than the median PFS of 16.6 months in the placebo/bevacizumab group (HR 0.59; 95% CI 0.49–0.72; *p* < 0.001). In the subgroup of patients with HRD (n = 387), the PFS for the olaparib/bevacizumab group was even longer, with a median of 37.2 months, compared to 17.7 months in the placebo/bevacizumab group (HR 0.33; 95% CI 0.25–0.45). The absence of the olaparib-only maintenance group was limitation of this trial.

In an ATHENA (GOG-3020/ENGOT-ov45 trial [7], rucaparib as first-line maintenance in ovarian cancer patients was evaluated. A total of 538 patients with newly diagnosed, histologically confirmed, advanced (stage III–IV), high-grade epithelial ovarian cancer who had completed cytoreductive surgery (R0/complete resection was permitted) before chemotherapy or following neoadjuvant were enrolled for investigation. Patients were randomized 4:1 to receive rucaparib or a placebo as maintenance therapy. Median PFS was 28.7 months with rucaparib versus 11.3 months with placebo in the HRD population (HR 0.47; 95% CI, 0.31 to 0.72, *p* = 0.0004); 20.2 months versus 9.2 months in the intent-to-treat population (HR 0.52; 95% CI, 0.40 to 0.68; *p* < 0.0001) and 12.1 months versus 9.1 months in the HRD-negative population (HR 0.65; 95% CI 0.45 to 0.95).

## 4. HRD Assays in Ovarian Cancer RCTs and HRD Prevalence

HRD assays are intended to predict HRD based on genomic characteristics, and are needed for patient selection for PARP inhibitor in ovarian cancer patients. HRD diagnostic tests examine various components, resulting in different HR status definitions and, as a result, different treatment decisions [22]. The Myriad myChoice CDx and FoundationOne CDx tests are approved by FDA, and were used in PARP inhibitor trials. For therapy with olaparib and niraparib, the Myriad myChoice CDx test was approved as a companion diagnostic method [5,21]. To determine the HRD score threshold, Myriad HRD scores were evaluated in a training group of 497 breast and 561 ovarian chemotherapy-naive cancers with known *BRCA1/2* status. A cut-off was established that had a 95% sensitivity for detecting tumors with *BRCA1/2* mutations or *BRCA1* promoter methylation [23]. Tumors with *BRCA1/2* deficiency have high genomic instability scores (GIS), and the threshold was set at 42 to identify HRD tumors with 95% sensitivity. It is worth noting that some tumors with unaltered *BRCA1/2* may also have elevated GIS due to non-*BRCA1/2* HRR gene alterations [24]. To improve the detection of responses to PARP inhibitors, the GIS cutoff was recently lowered from 42 to 33 in some cases [6]. In the rucaparib trial [7], an extensive panel of genes, including *BRCA*1/2 and other HRR genes, was analyzed using the FoundationOne CDx [25]. The assay determines HR status by combining the percentage of LOH regions in the tumor genome with *BRCA*1/2 alterations. Next-generation sequencing was used to determine the rate of genomic LOH, and LOH-high is defined as exceeding the 14% cutoff [8].

In the results of trials shown in Table 1, the *BRCA* mutation prevalence was 22% to 30% and the HRD prevalence was 43 to 55%. In PRIMA [5], VELIA [6], and PAOLA-1 [21], Myriad myChoice test was used as the central testing method, but there was a difference in HRD definition. In VELIA, the HRD score cutoff was set at 33 to increase sensitivity, which is different from other RCTs using 44 as a HRD cutoff. The fact that the HRD prevalence in VELIA is 55%, which is higher than in other studies, may be a related to a different HRD cutoff score. In the ATHENA [7] study, Foundation One CDx assay was used, and this may have led to the lower HRD prevalence in included patients.

## 5. Subset Data/Real-World Data of PARP Inhibitors Maintenance RCTs and HRD Prevalence

Subset data of PARP inhibitors maintenance RCTs are shown in Table 2. These subset data have the disadvantage of a small sample size, and as a result, statistical significance may not be demonstrated, and it is necessary to take this into consideration when interpreting the results. In the subset data of the PARP inhibitor maintenance trials, there was a difference in HRD prevalence by race in ovarian cancer patients. In the Japanese subgroup data of the VELIA study [26], 36% of the *BRCA* mutation and 73% of the HRD prevalence are observed. This was higher than the non-Japanese subgroup with 62% in the VELIA study. In the Japanese subset data of the PAOLA-1 study [27], the HRD prevalence was 67%, which was higher than the HRD prevalence of the entire study population of PAOLA-1 (48%). In the PRIME study [28], a niraparib maintenance treatment trial in Chinese ovarian cancer patients, a *BRCA* mutation prevalence of 33% and an HRD prevalence of 67% were observed. Although these results are preliminary and not yet fully peer-reviewed and published, it is noteworthy that the HRD prevalence was higher in Chinese ovarian cancer patients than in the results of other multicenter studies with larger numbers of included patients. It should be taken into consideration that there were differences in diagnostic test methods between trials. It remains to be seen whether the results of this study will be finally confirmed in the future. In addition, in the real-world data of Chinese ovarian cancer patients who received PARP inhibitor treatment China [29], the HRD prevalence was 69%, which is also higher than result of other RCTs.

HRD prevalence in Japanese and Chinese ovarian cancer patients tended to be higher than in the entire trial patient group. Considering the small number of patients or differences in diagnostic methods in these subset data, higher HRD prevalence in Asian ovarian cancer patients should be noted, and further large-scale investigations on racial differences in HRD prevalence are needed. The higher prevalence of HRD in ovarian cancer patients in Asia (including Japan and China) means the more patients who can benefit from PARP inhibitors, which may affect the treatment plan or possibly modifications in treatment guidelines in these patient group.

## 6. Conclusions

In the treatment of ovarian cancer, the efficacy of PARP inhibitor maintenance in HRD patients has been demonstrated through several clinical trials. The results of HRD tests conducted during the treatment of ovarian cancer patients are used as biomarkers for treatment and are used as criteria for determining adequate treatment. There is the possibility that certain patient groups may or may not be indicated for PARP inhibitor depending on the diagnostic test company, method, and cutoff. This may affect the prognosis of ovarian cancer patients. In planning ovarian cancer treatment, it is important to select an appropriate method that can present an accurate HRD phenotype of cancers to predict sensitivity to PARP inhibitors so that patients who are most likely to benefit from treatment could be selected.

In subset data of Asian patients of PARP inhibitor RCTs, higher HRD prevalence was observed. Additional research is needed to demonstrate differences in HRD prevalence between races, and these differences may affect future treatment guidelines or study design modifications. If the difference of HRD prevalence in a certain patient group is objectively proven, this may contribute to changes in determining the treatment plan and personalized treatment in ovarian cancer patients in the future.

## Figures and Tables

**Table 1:** Representative RCTs of PARP inhibitor first line maintenance in ovarian cancer patients with HRD.

	Study Design	Maintenance Therapy/PARP Inhibitor	Inclusion Criteria	Stratification Factors	Primary Endpoint	Results	*BRCA* Mutation Prevalence	HRD Prevalence	HRD Definition	Central Testing Method
PRIMA/ENGOT-OV26/GOG-3012 (NCT02655016) [5]	Randomized, double-blind, placebo-controlled phase 3 trial, a 2:1 ratio to receive niraparib or placebo once daily after a response to platinum-based chemotherapy	niraparib	Newly diagnosed, histologically confirmed advanced cancer of the ovary, high-grade, stage III or IV, stage III disease with visible residual tumor after primary debulking surgery, inoperable stage III disease, or any stage IV disease, as well as those who had received neoadjuvant chemotherapy	Myriad tumor HRD status cut-off 42	PFS	HRD cohort, HR 0.43; 95% CI 0.31–0.59; *p* < 0.001 Overall population HR 0.62; 95% CI 0.50 to 0.76; *p* < 0.001	30% (223/733)	50.9% (373/733)	the presence of a *BRCA* deleterious mutation, a score of at least 42 on the my Choice test, or both.	Myriad myChoice test
VELIA/GOG-3005 trial (NCT02470585) [6]	Randomly assigned in a 1:1:1 ratio to receive chemotherapy plus placebo followed by placebo maintenance (control), chemotherapy plus veliparib followed by placebo maintenance (veliparib combination only), or chemotherapy plus veliparib followed by veliparib maintenance (veliparib throughout)	veliparib	Initial histologic diagnosis of high-grade serous epithelial ovarian, carcinoma of stage III or IV	*BRCA* mutation status, Myriad tumor HRD status cut-off 33	Investigator-assessed PFS	*BRCA*-mutation cohort HR 0.44; 95% CI 0.28 to 0.68; *p* < 0.001. HRD cohort HR 0.57; 95% CI 0.43 to 0.76; *p* < 0.001 Intention-to treat population, HR 0.68; 95% CI 0.56 to 0.83; *p* < 0.001	26% (298/1140), -g*BRCA*m 19% -s*BRCA*m 7%	55% (627/1140) -*BRCA*m 26% -non *BRCA*m 29%	a score of ≥33 was considered to indicate HRD status, and a score of <33 was considered to indicate non-HRD status the threshold score was revised from 42, after several retrospective analyses of previous clinical trials, to increase the sensitivity of detecting a response to PARP inhibitors	Myriad BRACAnalysis CDx or myChoice HRD CDx assay
PAOLA-1/ENGOT-ov25 trial (NCT02477644) [21]	Randomly assigned in a 2:1 ratio to receive olaparib tablets (300 mg twice daily) or placebo for up to 24 months; all the patients received bevacizumab at a dose of 15 mg per kilogram of body weight every 3 weeks for up to 15 months in total.	olaparib + bevacizumab	Newly diagnosed, stage III or IV, high-grade serous or endometrioid ovarian cancer, and were having a response after first-line platinum–taxane chemotherapy	*BRCA* mutation status, Myriad tumor HRD status cut-off 42	Investigator-assessed PFS	*BRCA*-mutation cohort HR 0.31; 95% CI, 0.20 to 0.47. HRD cohort HR 0.33; 95% CI 0.25 to 0.45 Investigator-assessed, HR 0.59; 95% CI, 0.49 to 0.72; *p* < 0.001	30% (241/806)	48% (387/806)	HRD score of ≥42 (positive test) provided evidence of defects in homologous recombination	Myriad myChoice^®^ HRD Plus assay
ATHENA/GOG-3020/ENGOT-ov45 (NCT03522246) [7]	Randomly assigned 4:1 to oral rucaparib + intravenous (IV) placebo or oral placebo + IV placebo	rucaparib	Newly diagnosed, histologically confirmed, advanced (stage III-IV), high-grade epithelial ovarian cancer who had completed cytoreductive surgery (R0/complete resection was permitted) before chemotherapy or following neoadjuvant chemotherapy; and achieved an investigator-assessed response	HRD classification (*BRCA* mutation, *BRCA* wild-type/LOH high [LOH > 16%], *BRCA* wild-type/LOH low [LOH < 16%], and *BRCA* wild-type/LOH indeterminate), disease status after chemotherapy, timing of surgery	Investigator-assessed PFS	HRD cohort, HR 0.47; 95% CI 0.31 to 0.72; *p* = 0.0004. Intend-to-treat population HR 0.52; 95% CI 0.40 to 0.68; *p* < 0.0001	22% (115/538)	43% (234/538)	*BRCA*-mutant or *BRCA* wild-type/LOH high carcinoma	FoundationOne CDx next-generation sequencing assay

RCT: randomized-controlled trial, PARP: poly (ADP-ribose) polymerase, HRD: homologous recombination deficiency, PFS: progression-free survival, *BRCA*m: *BRCA* mutation, LOH: loss of heterozygosity.

**Table 2:** Results of subset data and real-world data from PARP inhibitor maintenance treatment trials in ovarian cancer patients.

	Study Design	Number of Patients	Results	*BRCA* Mutation Prevalance	HRD Prevalence	Central Testing Method
VELIA Japan subgroup [26]	Subgroup data of VELIA study	n = 78 - control (n = 23) - veliparib-combination-only (n = 30) - veliparib-throughout (n = 25)	In Japan subgroup, median progression-free survival was 27.4 months in the veliparib-throughout arm compared with 19.1 months in the control arm (HR, 0.46; 95% CI 0.18–1.16; *p* = 0.1 [not significant]).	36% (17/47) vs. non-Japanese subgroup 28% (183/652) *	73% (33/45) vs. non-Japanese subgroup 62% (388/625) *	Myriad BRACAnalysis CDx or myChoice HRD CDx assay (HRD score of ≥33)
Japan subset from the PAOLA-1/ENGOT-ov25 trial [27]	Subgroup data of PAOLA-1 study	n = 24 - Olaparib + bevacizumab (n = 15) - Placebo + bevacizumab (n = 9)	Investigator-assessed PFS was significantly longer in the olaparib plus bevacizumab group than in the placebo plus bevacizumab group (median 27.4 vs. 19.4 months; HR = 0.34; 95% CI 0.11–1.00)	21% (5/24)	67% (16/24)	Myriad myChoice^®^ HRD Plus assay (score of ≥42)
PRIME (NCT03709316), China [28]	Double-blind, placebo-controlled, multi-center phase 3 study - Chinese newly diagnosed, advanced (stage III–IV) high-grade serous or endometrioid ovarian cancer patients with response to chemotherapy - randomized 2:1 to niraparib or placebo	n = 384 - Niraparib n = 255 - Placebo n = 129	Intended-to-treat population, median PFS: 24.8 vs. 8.3 months; HR 0.45; 95% CI 0.34–0.60; *p* < 0.001 HRD population (HR 0.48)	33% (125/384)	67% (257/384)	BGI Genomics, Shenzhen, China
Real-world data, China, (NCT:05044091) ** [29]	Real-world data collected (2018~2021, data of ovarian cancer patients were treated with PAPRi for more than four weeks, including olaparib and niraparib in the Affiliated Cancer Hospital of Nanjing Medical University)	n = 67	PFS among HRD positive patients was significantly longer than those HRD negative patients (medium PFS 9.4 months vs. 4.1 months, HR: 0.52; 95% CI 0.38–0.71; *p* < 0.001)	36% (24/67)	69% (46/67)	AmoyDx^®^ HRD panel (HRD score ≥ 42 or BRCA mutation positive)

HRD: homologous recombination deficiency. * Some data on *BRCA* mutation and HRD status were missing. ** Not all patients were first line maintenance treated.

## Data Availability

No new data were created or analyzed in this study. Data sharing is not applicable to be this article.

## References

[1] Sung H., Ferlay J., Siegel R.L., Laversanne M., Soerjomataram I., Jemal A., Bray F. (2021). Global Cancer Statistics 2020: GLOBOCAN Estimates of Incidence and Mortality Worldwide for 36 Cancers in 185 Countries. CA Cancer J. Clin..

[2] Karam A., Ledermann J.A., Kim J.W., Sehouli J., Lu K., Gourley C., Katsumata N., Burger R.A., Nam B.H., Bacon M. (2017). Fifth Ovarian Cancer Consensus Conference of the Gynecologic Cancer InterGroup: First-line interventions. Ann. Oncol..

[3] Perren T.J., Swart A.M., Pfisterer J., Ledermann J.A., Pujade-Lauraine E., Kristensen G., Carey M.S., Beale P., Cervantes A., Kurzeder C. (2011). A phase 3 trial of bevacizumab in ovarian cancer. N. Engl. J. Med..

[4] Moore K., Colombo N., Scambia G., Kim B.G., Oaknin A., Friedlander M., Lisyanskaya A., Floquet A., Leary A., Sonke G.S. (2018). Maintenance Olaparib in Patients with Newly Diagnosed Advanced Ovarian Cancer. N. Engl. J. Med..

[5] González-Martín A., Pothuri B., Vergote I., DePont Christensen R., Graybill W., Mirza M.R., McCormick C., Lorusso D., Hoskins P., Freyer G. (2019). Niraparib in Patients with Newly Diagnosed Advanced Ovarian Cancer. N. Engl. J. Med..

[6] Coleman R.L., Fleming G.F., Brady M.F., Swisher E.M., Steffensen K.D., Friedlander M., Okamoto A., Moore K.N., Efrat Ben-Baruch N., Werner T.L. (2019). Veliparib with First-Line Chemotherapy and as Maintenance Therapy in Ovarian Cancer. N. Engl. J. Med..

[7] Monk B.J., Parkinson C., Lim M.C., O’Malley D.M., Oaknin A., Wilson M.K., Coleman R.L., Lorusso D., Bessette P., Ghamande S. (2022). A Randomized, Phase III Trial to Evaluate Rucaparib Monotherapy as Maintenance Treatment in Patients With Newly Diagnosed Ovarian Cancer (ATHENA-MONO/GOG-3020/ENGOT-ov45). J. Clin. Oncol..

[8] (2011). Integrated genomic analyses of ovarian carcinoma. Nature.

[9] Eoh K.J., Kim H.M., Lee J.Y., Kim S., Kim S.W., Kim Y.T., Nam E.J. (2020). Mutation landscape of germline and somatic BRCA1/2 in patients with high-grade serous ovarian cancer. BMC Cancer.

[10] Zhang H., Liu T., Zhang Z., Payne S.H., Zhang B., McDermott J.E., Zhou J.Y., Petyuk V.A., Chen L., Ray D. (2016). Integrated Proteogenomic Characterization of Human High-Grade Serous Ovarian Cancer. Cell.

[11] Yamamoto H., Hirasawa A. (2021). Homologous Recombination Deficiencies and Hereditary Tumors. Int. J. Mol. Sci..

[12] O’Connor M.J. (2015). Targeting the DNA Damage Response in Cancer. Mol. Cell..

[13] Ngoi N.Y.L., Tan D.S.P. (2021). The role of homologous recombination deficiency testing in ovarian cancer and its clinical implications: Do we need it?. ESMO Open.

[14] Abkevich V., Timms K.M., Hennessy B.T., Potter J., Carey M.S., Meyer L.A., Smith-McCune K., Broaddus R., Lu K.H., Chen J. (2012). Patterns of genomic loss of heterozygosity predict homologous recombination repair defects in epithelial ovarian cancer. Br. J. Cancer.

[15] Birkbak N.J., Wang Z.C., Kim J.Y., Eklund A.C., Li Q., Tian R., Bowman-Colin C., Li Y., Greene-Colozzi A., Iglehart J.D. (2012). Telomeric allelic imbalance indicates defective DNA repair and sensitivity to DNA-damaging agents. Cancer Discov..

[16] Popova T., Manié E., Rieunier G., Caux-Moncoutier V., Tirapo C., Dubois T., Delattre O., Sigal-Zafrani B., Bollet M., Longy M. (2012). Ploidy and large-scale genomic instability consistently identify basal-like breast carcinomas with BRCA1/2 inactivation. Cancer Res..

[17] Helleday T., Petermann E., Lundin C., Hodgson B., Sharma R.A. (2008). DNA repair pathways as targets for cancer therapy. Nat. Rev. Cancer.

[18] Denkert C., Romey M., Swedlund B., Hattesohl A., Teply-Szymanski J., Kommoss S., Kaiser K., Staebler A., du Bois A., Grass A. (2022). Homologous Recombination Deficiency as an Ovarian Cancer Biomarker in a Real-World Cohort: Validation of Decentralized Genomic Profiling. J. Mol. Diagn..

[19] Robson M.E., Tung N., Conte P., Im S.A., Senkus E., Xu B., Masuda N., Delaloge S., Li W., Armstrong A. (2019). OlympiAD final overall survival and tolerability results: Olaparib versus chemotherapy treatment of physician’s choice in patients with a germline BRCA mutation and HER2-negative metastatic breast cancer. Ann. Oncol..

[20] de Bono J., Mateo J., Fizazi K., Saad F., Shore N., Sandhu S., Chi K.N., Sartor O., Agarwal N., Olmos D. (2020). Olaparib for Metastatic Castration-Resistant Prostate Cancer. N. Engl. J. Med..

[21] Ray-Coquard I., Pautier P., Pignata S., Pérol D., González-Martín A., Berger R., Fujiwara K., Vergote I., Colombo N., Mäenpää J. (2019). Olaparib plus Bevacizumab as First-Line Maintenance in Ovarian Cancer. N. Engl. J. Med..

[22] Stewart M.D., Merino Vega D., Arend R.C., Baden J.F., Barbash O., Beaubier N., Collins G., French T., Ghahramani N., Hinson P. (2022). Homologous Recombination Deficiency: Concepts, Definitions, and Assays. Oncologist.

[23] Telli M.L., Timms K.M., Reid J., Hennessy B., Mills G.B., Jensen K.C., Szallasi Z., Barry W.T., Winer E.P., Tung N.M. (2016). Homologous Recombination Deficiency (HRD) Score Predicts Response to Platinum-Containing Neoadjuvant Chemotherapy in Patients with Triple-Negative Breast Cancer. Clin. Cancer Res..

[24] Stronach E.A., Paul J., Timms K.M., Hughes E., Brown K., Neff C., Perry M., Gutin A., El-Bahrawy M., Steel J.H. (2018). Biomarker Assessment of HR Deficiency, Tumor BRCA1/2 Mutations, and CCNE1 Copy Number in Ovarian Cancer: Associations with Clinical Outcome Following Platinum Monotherapy. Mol. Cancer Res..

[25] Paulet L., Trecourt A., Leary A., Peron J., Descotes F., Devouassoux-Shisheboran M., Leroy K., You B., Lopez J. (2022). Cracking the homologous recombination deficiency code: How to identify responders to PARP inhibitors. Eur. J. Cancer.

[26] Mizuno M., Ito K., Nakai H., Kato H., Kamiura S., Ushijima K., Nagao S., Takano H., Okadome M., Takekuma M. (2023). Veliparib with frontline chemotherapy and as maintenance in Japanese women with ovarian cancer: A subanalysis of efficacy, safety, and antiemetic use in the phase 3 VELIA trial. Int. J. Clin. Oncol..

[27] Fujiwara K., Fujiwara H., Yoshida H., Satoh T., Yonemori K., Nagao S., Matsumoto T., Kobayashi H., Bourgeois H., Harter P. (2021). Olaparib plus bevacizumab as maintenance therapy in patients with newly diagnosed, advanced ovarian cancer: Japan subset from the PAOLA-1/ENGOT-ov25 trial. J. Gynecol. Oncol..

[28] Li N., Zhu J., Yin R., Wang J., Pan L., Kong B., Zheng H., Liu J., Wu X., Wang L. (2022). Efficacy and safety of niraparib as maintenance treatment in patients with newly diagnosed advanced ovarian cancer using an individualized starting dose (PRIME Study): A randomized, double-blind, placebo-controlled, phase 3 trial (LBA 5). Gynecol. Oncol..

[29] Ni J., Guo W., Zhao Q., Cheng X., Xu X., Zhou R., Gu H., Chen C., Chen X. (2021). Homologous Recombination Deficiency Associated With Response to Poly (ADP-ribose) Polymerase Inhibitors in Ovarian Cancer Patients: The First Real-World Evidence From China. Front. Oncol..

